# A new frame shift pathogenic variant (c.773dupT) in the *BCKDHB* gene caused MSUD in an infant from north of Iran

**DOI:** 10.1515/crpm-2024-0052

**Published:** 2026-06-16

**Authors:** Hossein Jalali, Daniel Zamanfar, Maryam Rahimi, Hossein Mokhtari, Fatemeh Ghorbani, Mohammad Reza Mahdavi

**Affiliations:** Thalassemia Research Center, Hemoglobinopathy Institute, Mazandaran University of Medical Sciences, Sari, Iran; Sinaye Mehr Research Center, Mazandaran University of Medical Sciences, Sari, Iran; Amol Faculty of Paramedicine, Mazandaran University of Medical Sciences, Sari, Iran

**Keywords:** MSUD, *BCKDHB* gene, pathogenic variant, metabolic disorder

## Abstract

**Objectives:**

Inborn errors of metabolism (IEMs) result from pathogenic variants in genes involved in essential metabolic pathways. Newborn screening (NBS) using tandem mass spectrometry (MS/MS) has facilitated the early detection and diagnosis of IEMs, enabling timely medical intervention and improved clinical outcomes. This study introduces a novel pathogenic variant in the *BCKDHB* gene in a case of maple syrup urine disease (MSUD) diagnosed through the NBS program in northern Iran.

**Case presentation:**

Dried blood spot samples from newborns were analyzed using MS/MS (Shimadzu LCMS-8045, Japan), which identified a consanguineous case suspected of having MSUD. The confirmatory HPLC test revealed elevated levels of threonine, valine, isoleucine, and allo-isoleucine, indicating MSUD. Genetic analysis identified a homozygous frameshift variant, c.773dupT (NM_183050.4, p.Leu260ThrfsTer12), in exon 7 of the *BCKDHB* gene.

**Conclusions:**

The identification of novel pathogenic variants in MSUD patients, along with phenotype-genotype correlations, may predict the clinical severity of the variant and offer prognostic value, particularly for individuals with this specific variant.

## Introduction

Inborn errors of metabolism (IEMs) are inherited biochemical abnormalities caused by pathogenic variants in genes associated with essential metabolic pathways. These variants result in impaired enzyme function, disrupting the balance of vital metabolites [1]. The global prevalence of IEMs, which can lead to a variety of clinical symptoms depending on the underlying pathology, the location of the cellular defect, and the type of biological molecules involved, is estimated to be 2–3 % [2], 3].

Maple syrup urine disease (MSUD), first described in 1954, is a rare IEM characterized by an autosomal recessive inheritance pattern. It results from a reduced function of the branched-chain α-ketoacid dehydrogenase (BCKAD) enzyme complex. This deficiency leads to the accumulation of branched-chain amino acids (BCAAs), including leucine, isoleucine, and valine. BCAAs and their corresponding branched-chain keto acid (BCKA) metabolites accumulate in the brain, leading to a range of clinical manifestations, particularly neurological damage. Symptoms may include anorexia, apnea, and stereotyped movements, among others [4], 5]. Neonates with the classic form of MSUD have less than 2 % of BCKAD enzymatic activity, resulting in the characteristic maple syrup odor in urine within the first week of life [6].

The global incidence of MSUD is estimated to be approximately one in 185,000 live births [7]. With early diagnosis, treatment through dietary restriction of BCAAs can be initiated, significantly improving clinical outcomes. Consequently, MSUD is included in the recommended uniform screening panel (RUSP), which lists actionable, early-onset disorders recommended for newborn screening (NBS) in the United States [8].

NBS using tandem mass spectrometry (MS/MS) has facilitated the early detection and diagnosis of IEMs, enabling timely medical intervention that improves clinical outcomes in affected patients [8]. In 2018, NBS for metabolic disorders using MS/MS technology was introduced in certain provinces of Iran, and since then, several rare metabolic disorders have been reported in the country [9]. Mazandaran, a northern province of Iran located along the southern coastline of the Caspian Sea, has a relatively high incidence rate of MSUD, estimated at 1 in 27,714 live births [10]. Identifying novel variants responsible for the disease in each geographical region can contribute to a better understanding of its spread and pathogenicity.

The advent of novel sequencing technologies has enabled the identification of new pathogenic variants in the MSUD disease, which could be beneficial for families if prenatal testing is conducted in subsequent pregnancies [11]. The present study aims to introduce a novel pathogenic variant in the *BCKDHB* gene in a case of MSUD diagnosed through the NBS program in north of Iran.

## Case presentation

During the national NBS program for the detection of inborn errors of IEMs disorders, dried blood spot samples from newborns were referred to Fajr Medical Laboratory in Sari, Iran. The initial results of (MS/MS) test (Shimadzu LCMS-8045, Japan), which measures amino acid and acylcarnitine levels, identified a consanguineous case potentially affected by MSUD. Subsequently, a confirmatory HPLC test was performed on the neonate’s serum. The results revealed elevated levels of threonine, valine, isoleucine, and allo-isoleucine, which are indicative of MSUD (Table 1).

**Table 1:** Amino acids values in the patient with MSUD obtained by HPLC.

Amino acids	Concentration	Reference value, µmol/L
Asp	3.4	1–8
Glu	24.2	9–109
Ser	150.2	85–185
Gln	572.1	405–923
His	66.7	54–113
Gly	320.7	138–349
**Thr**	**271.7**	**59–195**
Cit	31.5	9–52
Arg	80.4	38–122
Ala	293.8	157–481
Tyr	83.9	31–108
Trp	51.6	30–94
Met	31.9	14–37
**Val**	**322**	**130–307**
Phe	46.8	38–86
**Ileu**	**181.6**	**33–97**
Leu	170.6	65–179
Orn	58.8	33–103
Lys	155.7	98–231
**Allo-isoleucine**	**101.7**	**<4**

The elevated amino acids are indicated as bold.

The results of the gas chromatography-mass spectrometry (GC-MS) test (Shimadzu QP2010 SE, Japan) on a urine sample indicated elevated levels of several metabolites, including 2-hydroxyisovaleric acid (225.4-fold), 2-ketoisocaproic acid (87-fold), 2-keto-3-methylvaleric acid (178.8-fold), 2-keto-isovaleric acid (153-fold), and 2-hydroxyisocaproic acid (22.8-fold). These findings are consistent with a diagnosis of MSUD in neonates. Treatment was initiated as soon as the GC-MS results became available on the fifth day after birth and the genetic test was subsequently conducted to identify the associated variant.

For genetic analysis of the gene responsible for the disease, whole exome sequencing (WES) was performed using the Illumina platform. The results revealed a homozygous frameshift variant, c.773dupT (NM_183050.4, p.Leu260ThrfsTer12), in exon 7 of the *BCKDHB* gene.

The identified variant has not been reported in the literature or in relevant databases such as the Human Gene Mutation Database (HGMD) or ClinVar. In silico analysis based on ACMG guidelines, using Franklin Genomics online software, categorized the variant as likely pathogenic. Additionally, the MutationTaster online prediction tool classified the variant as disease-causing. The effect of variant on protein, the population data were considered as criteria for predicting the pathogenicity of the variant.

**Figure 1:**
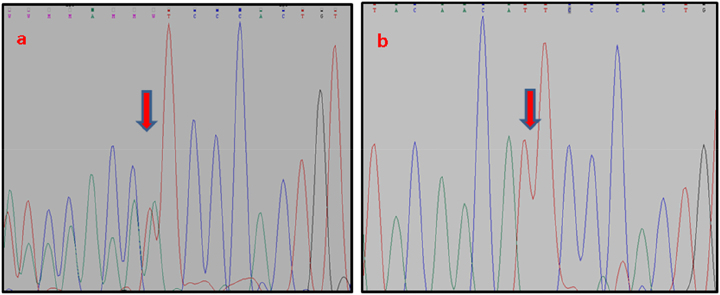
Sanger sequencing results of the case and his mother: (a) The homozygote case with c.773dupT variant. (b) The heterozygote mother with c.773dupT variant in the *BCKDHB* gene.

For segregation analysis of the detected variant, specific primers were designed for the target region of the *BCKDHB* gene (forward primer: 5-TCA​TCT​GTG​CAG​TAA​TGT​CAT​G-3, reverse primer: 5-AAA​TGA​GAG​CTT​CCA​AGC​AC-3). These primers were used to amplify the region containing the variant, and Sanger sequencing was performed using the 3130xl instrument (Applied Biosystems, USA) to detect the variant. The results showed the c.773dupT variant in a homozygous state in the case and in a heterozygous state in both parents (Figure 1).

## Discussion

Branched-chain amino acids (BCAAs), including valine, leucine, and isoleucine, make up approximately 40 % of the amino acids required by mammals. MSUD is a metabolic disorder caused by reduced function of the BCKAD enzyme complex. Homozygous pathogenic variants in the catalytic components of BCKAD reduce its activity, leading to elevated BCAA levels and causing toxicity in skeletal muscle and brain tissue. Proper BCAA catabolism is essential for normal physiological functions [8], 12], 13].

For the treatment of MSUD, a diet with limited BCAAs, providing only the minimum amount required for normal growth, is prescribed. Dietary treatment typically begins in newborns following a positive NBS result and confirmatory tests. Therefore, early diagnosis of the disease, as demonstrated in the present case, is crucial for initiating treatment and preventing the onset of clinical manifestations.

In the present study, we report a case of MSUD diagnosed through the national NBS program for IEMs. Due to the high rate of consanguineous marriages in Iran, a higher incidence of autosomal recessive diseases, such as MSUD, is expected compared to other populations [14]. Therefore, the continuation of the national screening program for metabolic disorders is strongly recommended in Iran.

Abiri et al. investigated the spectrum of pathogenic variants in four genes (*BCKDHA*, *BCKDHB*, *DBT*, and *DLD*) responsible for MSUD in 40 consanguineous patients [14]. They reported that of the investigated family 23 had pathogenic variants in the *BCKDHB*, 12 in the *BCKDHA* gene, 5 in the *DBT* genes. They reported 10 novel variants in the investigated families and among the families with pathogenic variants in the *BCKDHB* gene four families carried novel pathogenic variants including c.484 A>G, c.834_836dup CAC, c.357del T, and c. (343 + 1_344–1) _ (742 + 1_743–1) Del. The genetic analysis of 5 MSUD patients in Lebanon indicated that three patients had pathogenic variants in the *DBT* gene, one in the *BCKDHA* gene, and one in the *BCKDHB* gene. They reported three novel pathogenic variants including two variants in the *DBT* gene (c.224G>A and c.1430T>G) and one in the *BCKDHA* gene (c.488_1167+3del) [15]. Lashkarian et al. have also reported a novel pathogenic variant in the *BCKDHB* gene (c.552_553insA) of the 7 years old patient from Iran [16]. In Southwest Iran among the 23 investigated MSUD cases 10 different pathogenic variants were identified three of which were located in the *BCKDHB* gene including c.496A>G, c.508C>T, and c.988G>A [17]. In the present study, we have reported a novel pathogenic variant, (c.773dupT), in the *BCKDHB* gene in a family from northern Iran, highlighting the diversity of pathogenic variants in the *BCKDHB* gene in the region.

The identification of novel pathogenic variants in MSUD patients aids in prenatal diagnosis (PND) and pre-implantation genetic diagnosis (PGD). Additionally, it may support the implementation of community-based carrier testing within the population.
